# Pentosan Polysulfate: A Novel Therapy for the Mucopolysaccharidoses

**DOI:** 10.1371/journal.pone.0054459

**Published:** 2013-01-24

**Authors:** Edward H. Schuchman, Yi Ge, Alon Lai, Yury Borisov, Meghan Faillace, Efrat Eliyahu, Xingxuan He, James Iatridis, Helen Vlassara, Gary Striker, Calogera M. Simonaro

**Affiliations:** 1 Genetics and Genomic Sciences, Mount Sinai School of Medicine, New York, New York, United States of America; 2 Orthopedics, Mount Sinai School of Medicine, New York, New York, United States of America; 3 Medicine and Geriatrics and Palliative Care, Mount Sinai School of Medicine, New York, New York, United States of America; Baylor Research Institute, United States of America

## Abstract

**Background:**

Pentosan polysulfate (PPS) is an FDA-approved, oral medication with anti-inflammatory and pro-chondrogenic properties. We have previously shown that animal models of the mucopolysaccharidoses (MPS) exhibit significant inflammatory disease, contributing to cartilage degeneration. Enzyme replacement therapy (ERT) only partly reduced inflammation, and anti-TNF-alpha antibody therapy significantly enhanced clinical and pathological outcomes. Here we describe the use of PPS for the treatment of MPS type VI rats.

**Methodology/Principal Findings:**

Treatment began during prenatal development and at 1 and 6 months of age. All animals were treated until they were 9 months old. Significant reductions in the serum and tissue levels of several inflammatory markers (e.g., TNF-alpha, MIP-1alpha and RANTES/CCL5) were observed, as was reduced expression of inflammatory markers in cultured articular chondrocytes. ADAMTS-5/aggrecanase-2 levels also were reduced in chondrocytes, consistent with an elevation of serum tissue inhibitor of metalloproteinase 1. Marked improvements in motility and grooming behavior occurred, along with a reduction in eye and nasal secretions and a lessening of the tracheal deformities. MicroCT and radiographic analyses further revealed that the treated MPS skulls were longer and thinner, and that the teeth malocclusions, misalignments and mineral densities were improved. MicroCT analysis of the femurs and vertebrae revealed improvements in trabecular bone mineral densities, number and spacing in a subset of treated MPS animals. Biomechanical assessments of PPS-treated spines showed partially restored torsional behaviors, suggesting increased spinal stability. No improvements were observed in cortical bone or femur length. The positive changes in the PPS-treated MPS VI rats occurred despite glycosaminoglycan accumulation in their tissues.

**Conclusions:**

Based on these findings we conclude that PPS could be a simple and effective therapy for MPS that might provide significant clinical benefits alone and in combination with other therapies.

## Introduction

The mucopolysaccharidoses (MPS) comprise a group of 11 distinct lysosomal storage disorders due to inherited deficiencies of enzymes involved in glycosaminoglycan (GAG) catabolism [1]–[3], resulting in severe skin, bone and joint abnormalities, spinal cord and tracheal defects, and cardiac valve disease. Central nervous system (CNS) abnormalities also occur in many MPS patients, and lifespan is invariably shortened.

There are currently two main therapeutic options for MPS [4]. The first is hematopoietic stem cell transplantation (HSCT), and to date hundreds of MPS patients have received such transplants with variable success. In part, the limited success relates to the variable engraftment efficiencies and the fact that the transplanted cells cannot reach many of the important pathological sites in MPS (e.g., articular cartilage, growth plates, brain). In addition, the transplant procedures are often associated with significant morbidity and/or mortality.

Another therapeutic approach involves replacement of the missing enzymes with their normal, recombinant counterparts (i.e., enzyme replacement therapy, ERT). This type of therapy is available for three of the MPS (MPS I, II and VI) and under development for several others. Significant quality-of-life improvements have been documented in MPS patients treated by ERT, including improvements in joint mobility and motility. However, the clinical experience with ERT also has been variable, and in general the effects on cartilage and bone are modest. In addition, the infused enzymes do not cross the blood brain barrier, and antibody responses to the recombinant enzymes occur in some patients.

Due to the above limitations, research has continued to focus on investigating the pathophysiology of the MPS diseases to identify new therapeutic targets. For the past several years our group has studied the mechanism of cartilage and bone disease in MPS animal models. These studies showed that GAG storage in MPS cells activates toll-like receptor 4 (TLR4) signaling, ultimately leading to the release of TNF-alpha and other inflammatory cytokines [5]–[7]. Apoptosis also occurs in some MPS connective tissue cells (e.g., articular chondrocytes), while in others (e.g., synovial fibroblasts) proliferation may result. Thus, we hypothesized that GAG-stimulated inflammation is an important and previously unrecognized feature of the skeletal pathology in MPS.

Proof-of-concept for this hypothesis was obtained by creating a double mutant mouse that had MPS type VII on a TLR4 knockout background [8]. In addition, we treated MPS VI rats using anti-TNF-alpha antibody therapy, revealing several benefits over ERT alone [8], [9]. However, while these findings were encouraging, anti-TNF-alpha antibody therapy would be difficult to implement in MPS patients due to the potential immunosuppressive side effects, high cost, and need for additional invasive treatments.

Pentosan polysulfate (PPS) is an FDA-approved, oral medication (Elmiron®) with potent anti-inflammatory effects that is used to treat patients with interstitial cystitis (IC) [10]. It also has been shown to promote chondrogenesis in animals with osteoarthritis (OA) [11]–[13], and has been used off-label to treat OA patients [14]–[15]. Sodium or calcium salts of PPS are effective at reducing joint inflammation, promoting fibrinolysis, stimulating hyaluronan synthesis by synovial fibroblasts, and stimulating proteoglycan synthesis by chondrocytes [16]–[17]. They also promote proliferation and chondrogenic differentiation of adult human bone marrow mesenchymal stem cells [18].

Here we document the anti-inflammatory and clinical effects of oral PPS treatment in the rat model of MPS VI, even in the absence of other therapies to reduce GAG storage. We propose that PPS could be a safe and cost-effective therapy that might be used in combination with ERT, HSCT or other therapies for all MPS patients to improve the clinical outcomes.

## Results

### PPS Treatment Reduces Inflammation and Improves Cartilage Integrity in MPS VI Rats

Three groups of MPS VI rats were treated with PPS beginning at 6 months (group 1), 1 month (group 2), or during prenatal development (group 3). All animals were treated until they reached 9 months of age. Only female rats were treated to avoid gender-specific variations. Treated rats received PPS in their drinking water at a dose of 4 mg/kg/day, similar to the dose used to treat adult woman with IC (Note that actual dose is based on the amount of water consumption and weight of individual rats; see Material and Methods).

**Figure 1:**
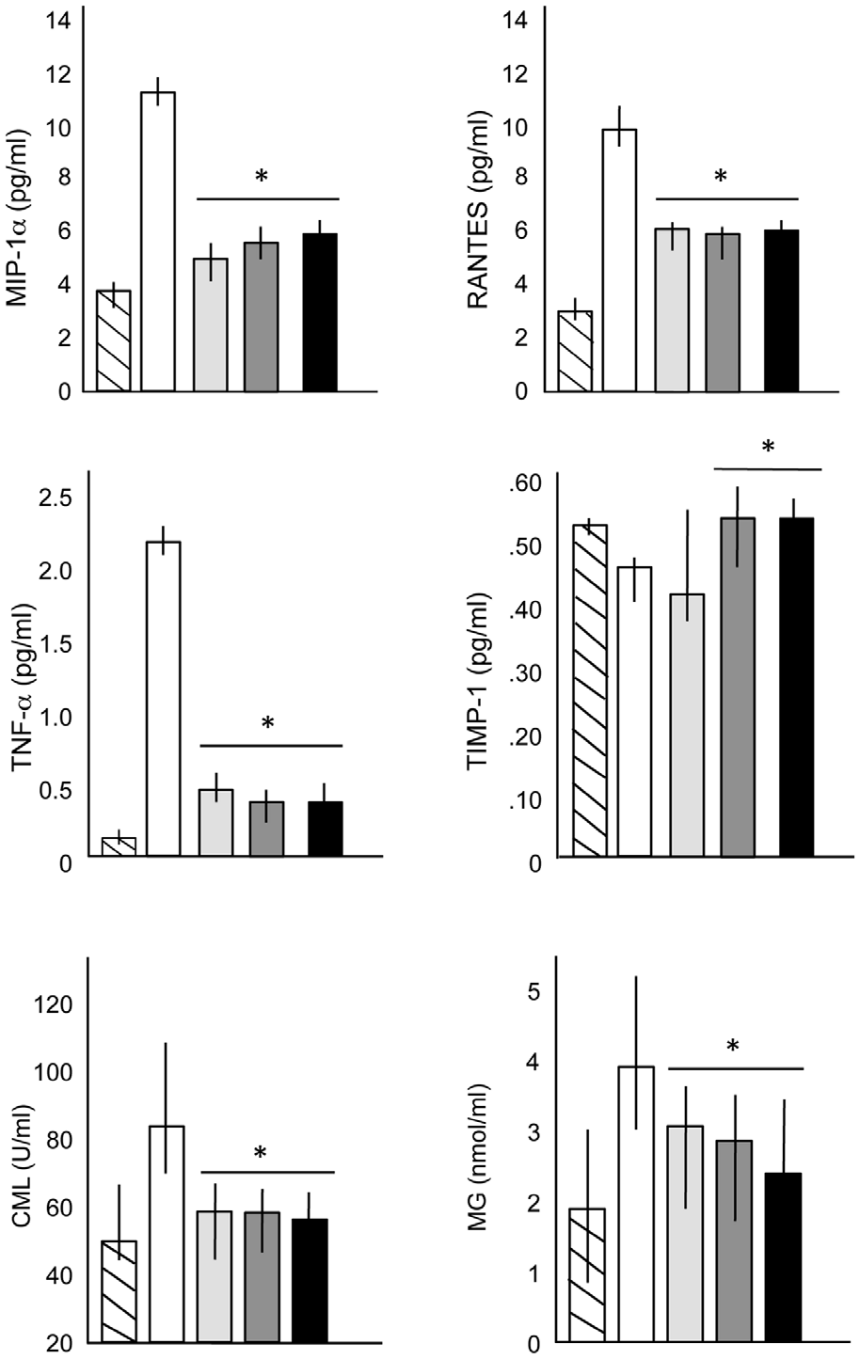
Serum assays in normal, untreated and PPS-treated MPS VI rats. Macrophage inflammatory protein-1alpha (MIP-1alpha), CCL5, regulated on activation normal T cell expressed and secreted (RANTES), tumor necrosis factor-alpha (TNF-alpha), tissue inhibitors of metalloproteinases-1 (TIMP-1), and the advanced glycation endproducts (AGEs; *N* ^e^-carboxymethyl-lysine (CML) and methyglyoxal (MG)), were quantified by ELISA assays as described in the Materials and Methods. Hatched boxes, normal rats; white boxes, untreated MPS VI rats; light gray boxes, group 1 treated MPS VI rats; dark gray boxes, group 2 treated MPS VI rats; and black boxes, group 3 treated MPS VI rats. N = 10/group. The vertical lines in each column indicate the ranges. *P<0.05 comparing treated to untreated MPS VI rats.

**Figure 2:**
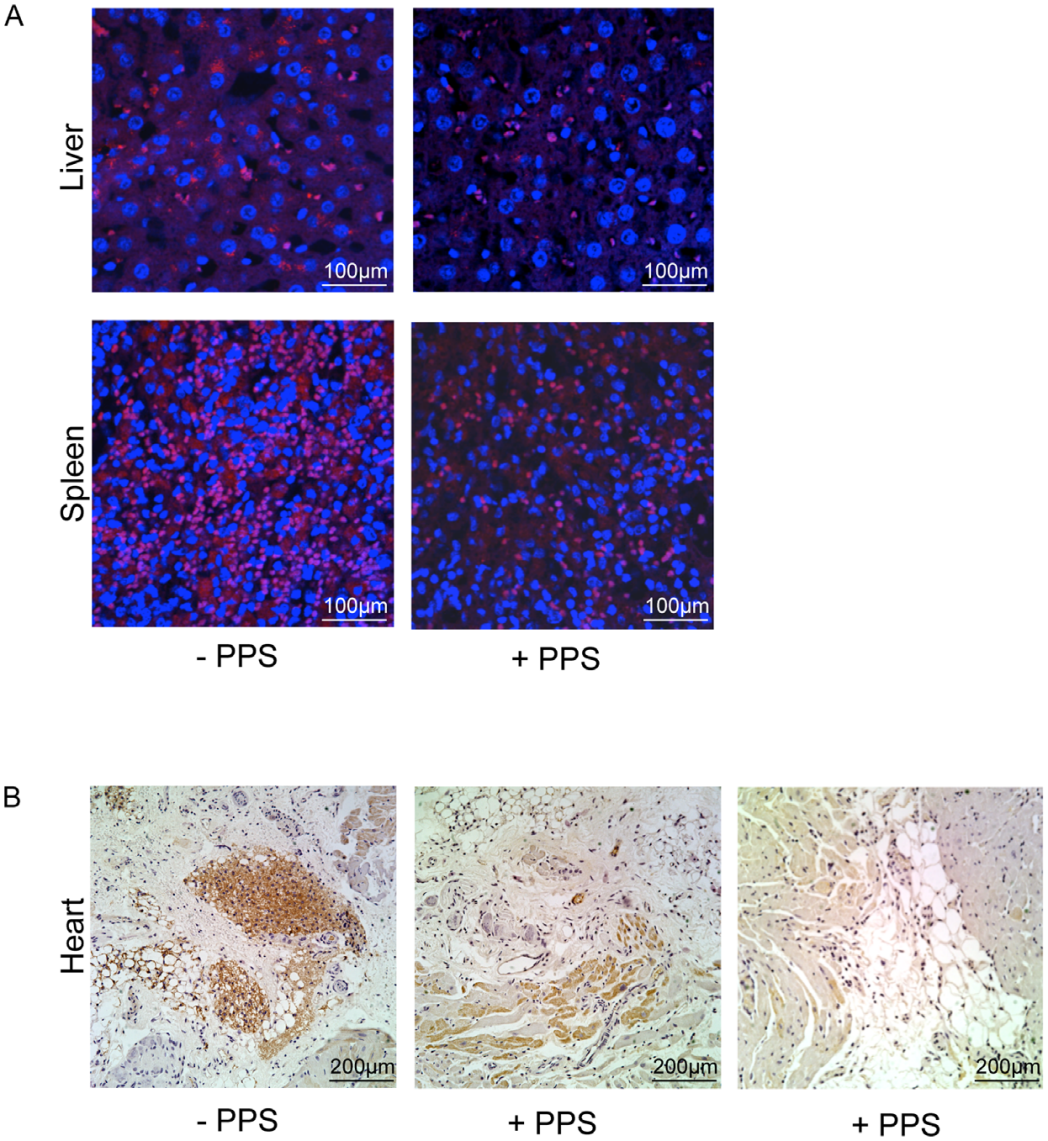
TNF-alpha immunohistochemistry. A) TNF-alpha was visualized in liver and spleen sections by fluorescent immunohistochemistry as described in the Materials and Methods. Representative images from one untreated and treated (group 2) MPS VI rat are shown. Red = TNF-alpha. Blue = DAPI. B) Light microscopy and DAB staining was used to visualize TNF-alpha in the heart. Brown color indicates positive staining. Representative images from one untreated and two treated group 2 MPS VI rats are shown. Images are representative of multiple sections from at least 3 rats in each group. Similar reductions in TNF-alpha staining were seen in all treatment groups.

Fig. 1 shows that the elevated levels of several serum inflammatory markers in the MPS VI rats were reduced to near normal levels in the PPS-treated animals. No significant differences were observed between the three treatment groups. Reduced TNF-alpha levels also were observed in the livers, spleens and hearts by immunohistochemistry (Fig. 2). Advanced glycan endproducts (AGEs), *N* ^e^-carboxymethyl-lysine (CML) and methyglyoxal (MG), are another class of inflammatory markers that were found for the first time to be elevated in untreated MPS VI rat serum, and also were significantly reduced in the PPS-treated animals (Fig. 1).

**Figure 3:**
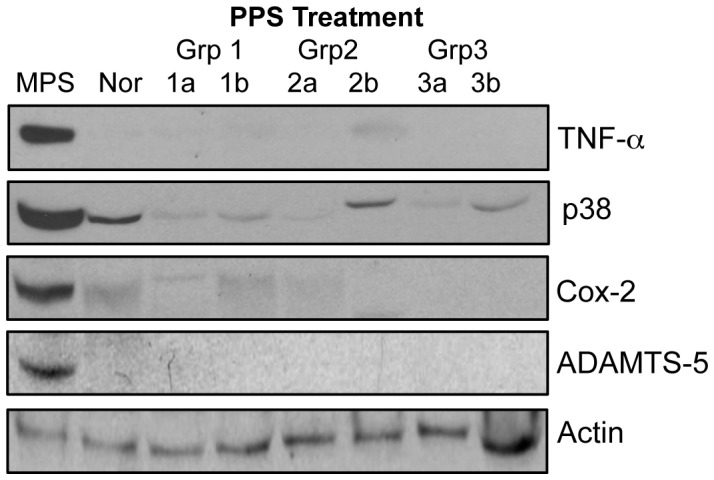
Western blot analysis of articular chondrocytes from PPS-treated and control rats. Articular chondrocytes were grown for 3 weeks as described in the Materials and Methods. Western blot analysis was then used to reveal the levels of TNF-alpha, p38, Cox-2 and ADAMTS-5. Each of these proteins was elevated in the untreated MPS VI rats (MPS) compared to normal (Nor) rats, and the levels were reduced to normal in the treated rats. Two animals each are shown for each of the three treatment groups. beta-actin was used as a loading control.

Articular chondrocytes were obtained at the end of the treatment period, grown for 3 weeks, and then analyzed by western blotting (Fig. 3). The expression of TNF-alpha, p38, and Cox-2, each of which are elevated in MPS VI rats [6]–[8], were reduced to normal levels in the PPS-treated animals. In addition, the levels of ADAMTS-5 (aggrecanase-2), which also is elevated in MPS VI rats, were reduced to normal. Consistent with this latter observation, the serum levels of tissue inhibitor of metalloproteinase 1 (TIMP-1), which are reduced in untreated MPS VI rats, were elevated following PPS treatment (Fig. 1).

**Figure 4:**
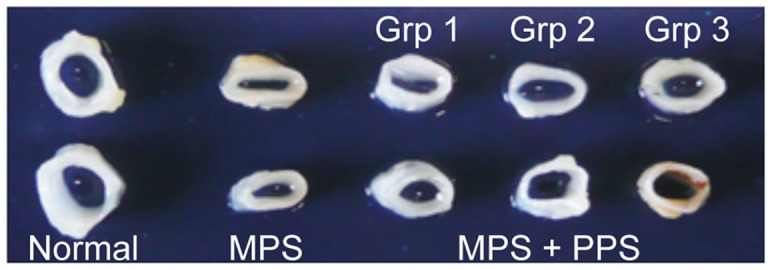
Tracheas from PPS-treated and control rats. Images of tracheas from normal, untreated MPS VI (MPS), and a representative animal from each PPS treatment group is shown. Note that the tracheas of untreated rats had thinner cartilage walls and smaller openings than normal rats, and that in the treated MPS VI rats the tracheal openings were wider.

To determine the effects of PPS treatment on another cartilaginous tissue, we also examined the integrity of the tracheas (Fig. 4). Untreated MPS VI rat tracheas are generally collapsed by 9 months. In contrast, tracheas from the PPS-treated MPS VI rats were more open and the cartilage walls were thicker. Together, these results indicated that PPS treatment was reducing inflammation and increasing both articular and hyaline cartilage integrity in the MPS VI animals.

### Increased Motility and Improved Grooming Behavior in Treated MPS VI Rats

**Figure 5:**
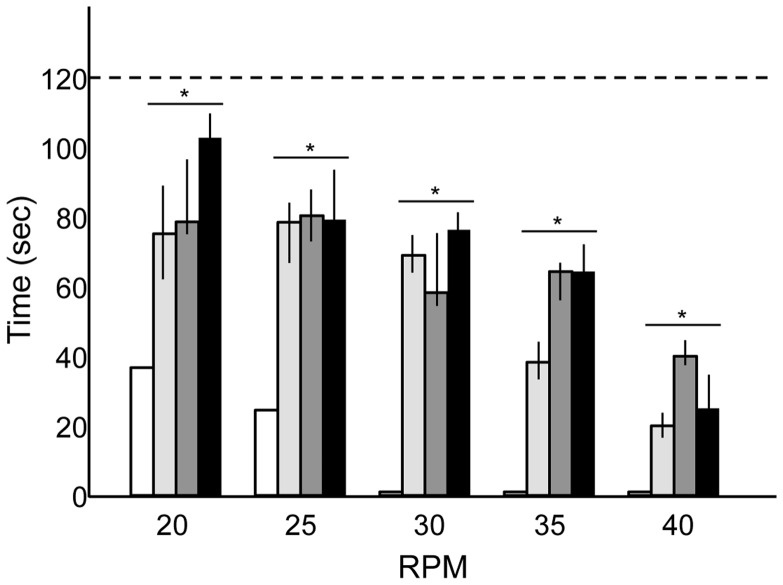
Rotarod analysis of untreated and PPS-treated MPS VI rats. Rotarod performance was assessed monthly in the untreated and PPS-treated MPS VI rats. Data is shown for all animals (n = 10/group) at 9 months of age, prior to sacrifice. White boxes, untreated MPS VI rats; light gray boxes, group 1 treated MPS VI rats; dark gray boxes, group 2 treated MPS VI rats; and black boxes, group 3 treated MPS VI rats. The vertical lines in each column indicate the ranges. The dotted horizontal line depicts the length of time that normal rats could remain on the rotarod at all speeds (120 s). In each of the treatment groups there was a significant improvement in the rotarod performance compared to untreated MPS VI rats (*P<0.05). This occurred at all speeds. Note that at or above 30 rpm the untreated MPS VI rats could not remain on the rotating bar.

MPS VI rats exhibited a markedly improved performance on an accelerating rotarod apparatus after PPS treatment (Fig. 5). This was evident at all speeds, but was most significant at or above 30 rpm, where the untreated MPS VI rats could not remain on the rotating bar. This behavior was further verified by observing the motility and appearance of the animals (see Movies S1, S2, and S3). As evident in these videos, treated MPS VI rats could stand on their hind limbs and were much more alert and mobile than their untreated littermates, and also had much smoother coats indicative of improved grooming.

### Skull and Dentition Changes in the PPS-treated MPS VI Rats

**Figure 6:**
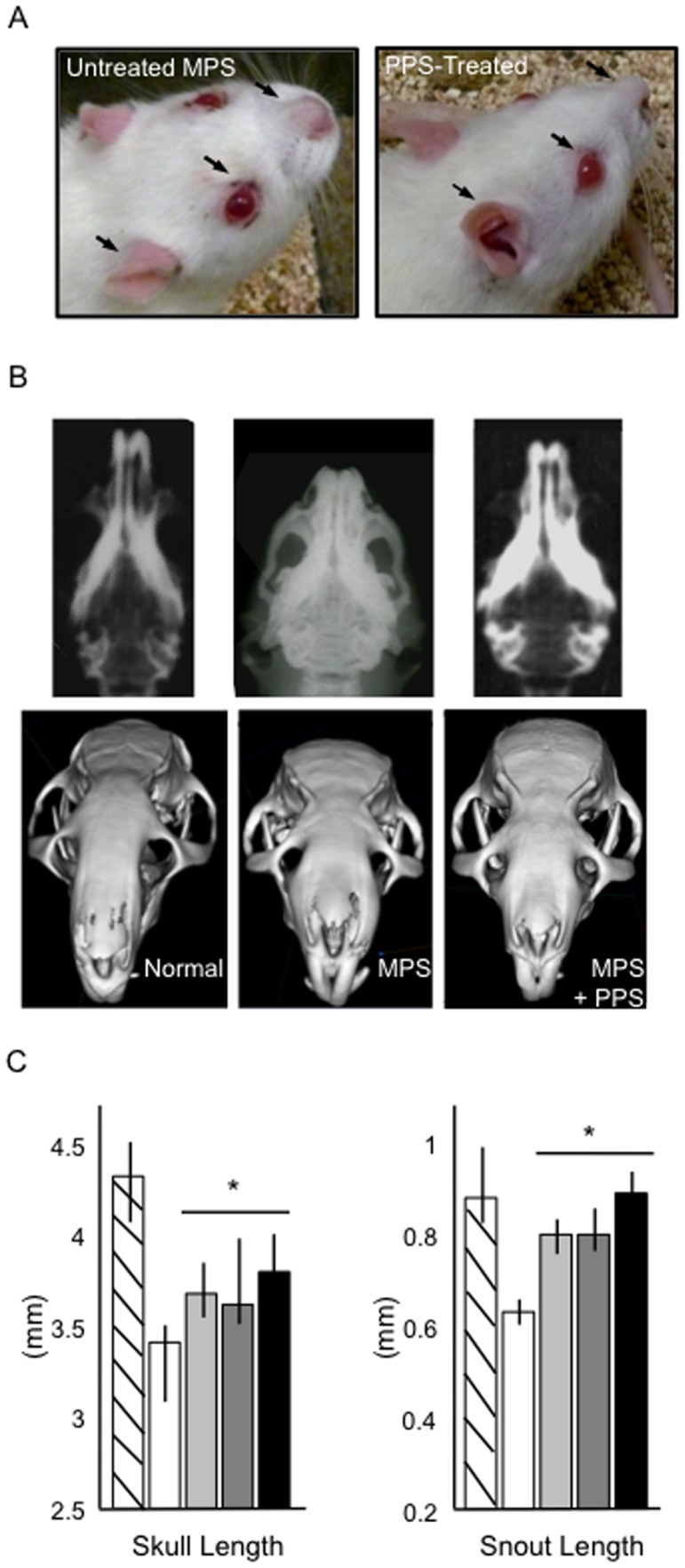
Skull changes in the PPS-treated MPS VI rats. A) Representative photographic images showing an untreated and PPS-treated (group 2) MPS VI rat. Black arrows indicate the eye and nasal secretions, which were reduced in the treated rats, and the ears, which were more erect in the treated animals. Also note the longer, thinner skulls. B) Representative radiograph and reconstructed microCT images of the skulls from a normal, untreated and treated MPS VI (group 2) rat. The longer and thinner snouts also are evident in these images. C) Quantitative radiographic measurements for all rats at 9 months of age. The skull and snouts lengths were statistically longer in all treated groups (*P<0.05). MicroCT reconstructions were carried out for at least 4 animals from each group.

MicroCT and/or radiographic analyses of the skulls were performed on 3–6 animals each from the control and PPS-treated MPS VI rat groups. Representative photographic images, radiographs and microCT reconstructions are shown in Fig. 6. Note the significantly longer skulls and snouts in the PPS-treated MPS VI rats compared to untreated MPS VI littermates. The skulls also were thinner following PPS treatment, although this did not reach statistical significance. Also evident in the images were the eye secretions that are typical of the MPS VI rats, and which were improved in the treatment groups. In addition, the improved grooming of the treated animals eluted to above and evident in the videos can be appreciated from these images, as well as the more erect and stiffer ears.

**Figure 7:**
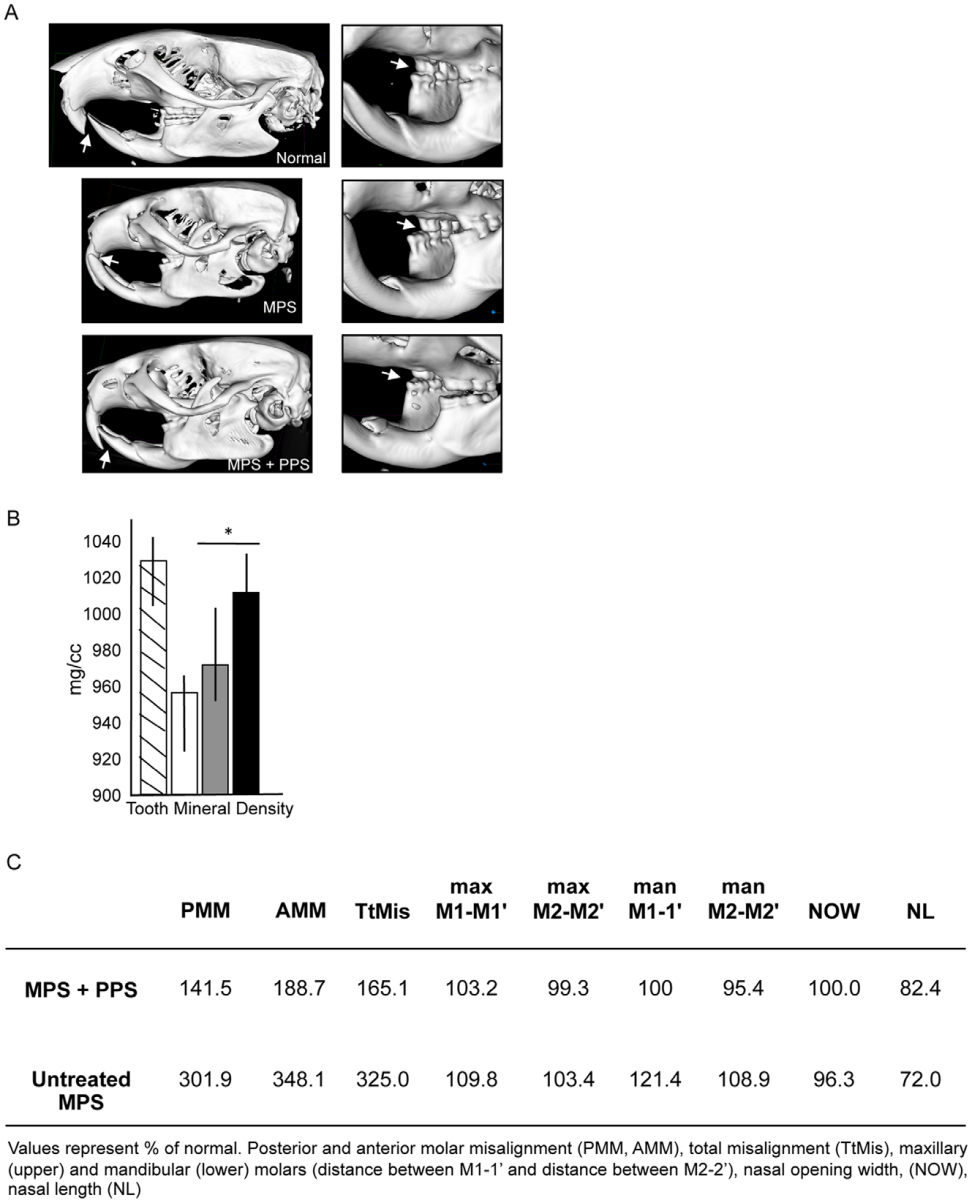
Dentition changes in the PPS-treated MPS VI rats. A) Reconstructed microCT images are shown for a representative normal, untreated MPS VI (MPS) and PPS-treated MPS VI (group 2) rat. The images to the right show a higher magnification to highlight the misalignment of the teeth in the untreated MPS VI rats (arrow), which was corrected in the treated animals. The skull microCT reconstructions were carried out for 6 rats each from groups 2 and 3, and similar observations were made. B) Tooth mineral densities (TMDs) were determined from the microCT analyses. Hatched boxes, normal rats; white boxes, untreated MPS VI rats; dark gray boxes, group 2 treated MPS VI rats; black boxes, group 3 treated MPS VI rats. Note that the TMDs were reduced in untreated rats compared to normal, and were significantly improved in each of the treatment groups (*P<0.05). C) Quantitative microCT measurements taken from reconstructed skull images from all of the analyzed PPS-treated group 2 and 3 rats (n = 12). Note the very significant posterior and anterior molar misalignment (PMM, AMM) evident in the untreated MPS VI rats, as well as the total misalignment (TtMis). In addition, the distance between the maxillary (upper) (maxM1–M1’ and maxM2–M2’) and mandibular (lower) (manM1–1′ and manM2–2′) molars in the untreated rats was wider than normal, and this was reduced in the treated rats. Finally, the nasal opening widths (NOW), which are moderately reduced in the untreated rats, were normalized in the treated rats, along with a ∼10% increase in nasal length (NL).

Other notable PPS improvements observed by microCT in the MPS VI rat skulls were apparent in the dentition, where malocclusions usually result due to the shift in the relationship of the maxillary and mandibular molars (Fig. 7). This misalignment prevents the natural grinding of the incisors and allows them to grow and curve, and results in trauma to the soft palate, infection and abscesses. In the PPS-treated animals a greater degree of alignment was seen between the manibular and maxillary molars, correcting the malocclusion. The tooth mineral densities also were significantly improved. Similar skull and dentition changes were observed in all of the treated animals, independent of when they were started on treatment or the duration.

### Femur and Spine Changes in the PPS-treated MPS VI Rats

MicroCT were performed on the femurs and L_2_ vertebrae from 6 animals each of the control and group 2 treated MPS VI rats. Tables 1 and 2 summarize the data. Of note, among the 6 PPS-treated animals there were 3 in which improvements were observed (“responders”), while little or no response was evident in the other 3 (“non-responders”). The same animals that showed changes in the femurs also exhibited changes in the vertebrae.

**Table 1:** Femur microCT analyses.

Trabecular	Tra BV/TV	TraTh	TraSpa	Tra#	TraBMD
MPS+PPS Responder	115.4	104.5	87.4	102.4	119
MPS+PPS Non Responder	56.2	82.4	160	68.5	73.8
PPS Combined Average	85.8	93.5	123.7	85.5	96.4
Untreated MPS	77.6	87.9	125	82.9	90.3
**Cortical**	**CtTh**	**CtArea**	**CtBMD**		
MPS+PPS Responder	127.7	103.5	105.4		
MPS+PPS Non Responder	133.9	109.3	103.4		
PPS Combined Average	130	106.4	102.7		
Untreated MPS	129.3	105.1	102.8		

Values represent % of normal. Tra = trabecular, BV/TV = bone volume/total volume, Th = thickness, Spa = space, # = number, BMD = bone mineral density.

Values represent % of normal. Ct = cortical, Th = thickness, BMD = bone mineral density.

**Table 2:** Vertebrae microCT analyses.

Trabecular	Tra BV/TV	TraTh		TraSpa		Tra#		TraTMD	
MPS+PPS Responder	84.5	100		131.8		83.4		81	
MPS+PPS Non Responder	61.6	84.2		177.4		79.9		61.7	
PPS Combined Average	73.1	92.1		154.6		81.6		71.4	
Untreated MPS	70.5	88.7		151.9		79.8		66.6	
**Cortical**	**CtTH**		**CtMar**		**CtTMD**		**CtArea**		
MPS+PPS Responder	90.9		77.2		102.5		77.5		
MPS+PPS Non Responder	84.4		73		103.4		76.5		
PPS Combined Average	87.7		75.1		102.7		77		
Untreated MPS	90.7		66.7		93.8		78.7		

Values represent % of normal. Tra = trabecular, BV/TV = bone volume/total volume, Spa = space, # = space, TMD = total mineral density.

Values represent % of normal. Ct = cortical, Mar = margin, TMD = tissue mineral density.

Overall, the trabecular abnormalities in untreated MPS VI rat femurs indicated an osteopenic phenotype with moderate rearrangement of the trabecular bone architecture, and this was improved in some of the PPS-treated animals. As summarized in Table 1, the most notable differences in the untreated MPS VI rat femurs were in the trabecular bone volume (BV/TV), which was ∼78% of normal, trabecular number (#), which was ∼83% of normal, and trabecular spacing (Spa), which was ∼125% of normal. Each of these parameters was improved in the responder group. Trabecular thickness (Th) and tissue mineral density (TMD) also were moderately reduced in the untreated MPS VI rat femurs, and improved in the responder group.

**Figure 8:**
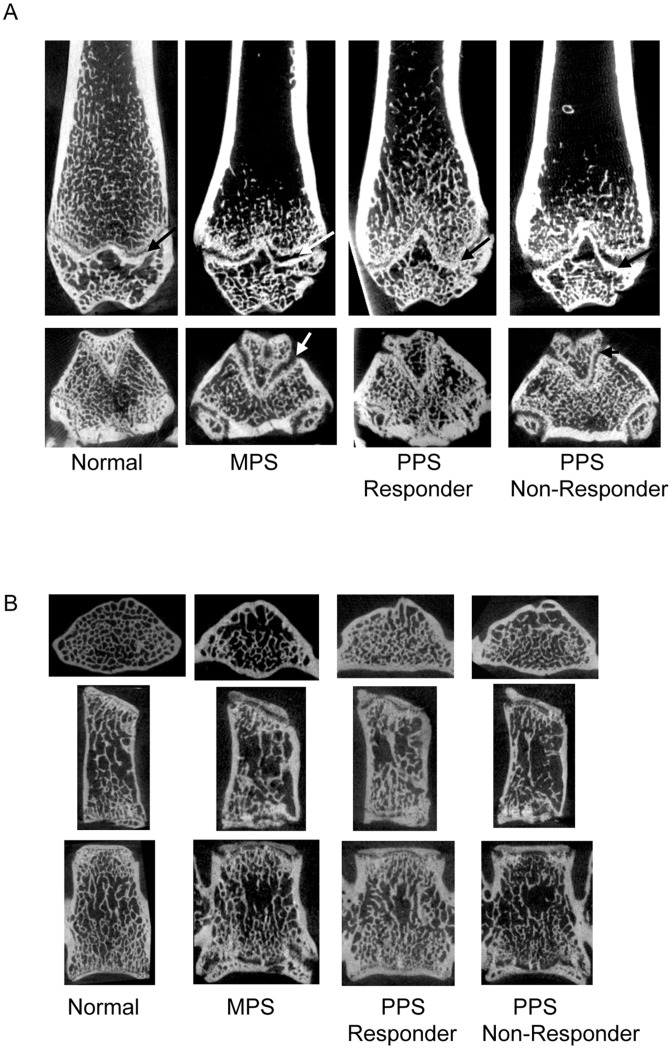
MicroCT images of femurs and vertebrae of PPS-treated and control rats. A) Representative reconstructed images from femurs showing mid-coronal and transverse growth plates (arrows). Note “detached” growth plates and condyles in MPS (white arrows) and non-responder animal (black arrows) which were improved in responder animals. B) Representative scaled 2D images from L2 vertebrae showing transverse, sagittal and coronal views. MPS vertebrae are shorter and wider than normal and are partially recovered with PPS treatment. Note that in the PPS-treated responder animal there were changes evident in the trabecular, but not in the cortical bone of both femurs and vertebrae.

MicroCT analysis also revealed thicker cortical bone in the untreated MPS VI rats (∼129% of normal), which was not improved in any of the treated MPS VI animals. The femurs also were shorter than normal (∼70%), and this was similarly not significantly improved by PPS treatment. Fig. 8A shows representative microCT images of the control and treated femurs for comparison.

Similar observations were made regarding the trabecular and cortical bone changes in the vertebrae of PPS-treated rats (Table 2). As in the femurs, trabecular abnormalities were observed in the untreated MPS VI vertebrae in all of the parameters, most notably in the spacing (Spa; ∼152% of normal), tissue mineral density (TMD; ∼67% of normal), and bone volume density (BV/TV; ∼71% of normal). Within the responder group positive changes were observed in the vertebrae in most parameters, most notably in the bone volume and tissue mineral density. Trabecular thickness also was normalized, and changes were evident in the trabecular spacing. There also was an increase in the vertebrae heights (from ∼68% to 75% of normal). Similar to the femurs, no significant improvements in the cortical bone were observed. Fig. 8B shows representative scaled microCT images of control and PPS-treated vertebrae for comparison.

Biomechanical assessments also were performed on the spines of the treated and control animals. Compared to the normal spine motion segment, there was an increased torsional neutral zone (NZ) length (137.8% of normal), as well as decreased NZ apparent modulus (51.5% of normal), apparent modulus (28.5% of normal) and torque range (15.3% of normal) in the untreated MPS VI rats. In all PPS treated groups there was a modest decrease in the NZ length and increase in the NZ apparent modulus relative to MPS VI, and the effects of PPS generally increased with duration of treatment. Failure load and stiffness of the untreated MPS VI vertebrae also were significantly lower than those of normal vertebra, and these were slightly improved following treatment.

## Discussion

Three groups of MPS VI rats were treated with PPS at a dose that approximated the human dose used to treat IC. PPS is supplied as a calcium or sodium salt, and the drug used in this study was a sodium salt obtained from Swati Spentose PVT. LTD. (India). Elmiron® (FDA-approved for IC) also is a sodium salt (manufactured by Bene Pharmachem, Germany), and the biological effects of the various preparations are reportedly equivalent [21].

Several serum inflammatory markers are highly elevated in MPS VI rats and other MPS animal models [5]–[7], and were significantly reduced in the PPS-treated animals. This included TNF-alpha, MIP-1alpha and RANTES/CCL5, all components of the TLR4 signaling pathway. We also report that AGEs, another class of inflammation markers that trigger oxidative stress and other damage [22]–[24], are elevated in the serum of MPS VI rats, and were significantly reduced by treatment. Overall, these findings are in accordance with the known anti-inflammatory properties of PPS, and show that it is effective even in animals with GAG storage, which is known to stimulate the inflammatory response (Fig. S1).

TNF-alpha, Cox-2 and p38 also are components of the TLR4 signaling pathway that are normally elevated in articular chondrocytes from MPS VI animals, and each were reduced to normal in the treated rat cells. Moreover, ADAMTS-5/aggrecanase-2 was decreased to normal, consistent with the elevated serum TIMP expression. Unfortunately, we could not quantify aggrecan directly in these cells since they were expanded for 3 weeks in order to obtain enough cells for western blotting, and cell expansion results in markedly reduced aggrecan expression [25].

TIMPs are reduced in OA, MPS and other inflammatory bone diseases, resulting in elevated activities of matrix-degrading MMPs and aggrecanases [6], [26]. PPS has been shown to increase the affinity between at least one aggrecanase (ADAMTS-5) and TIMP-3 in vitro [27], resulting in reduced aggrecanase activity. Previous animal studies by the same group [28] also showed that PPS elevated levels of cartilage TIMPs, leading to reduced levels of ADAMTS-4 and 5. Thus, our findings of enhanced TIMPs in the PPS-treated MPS VI rats are in accordance with these previous observations.

To assess the effect of PPS on another cartilaginous tissue we examined the tracheas, which usually collapse in untreated MPS VI rats. In contrast, in the PPS-treated animals the cartilage walls were thicker and the tracheas more open. Articular cartilage and tracheas represent two distinct types of cartilage, and the positive changes we observed in both tissues indicate the broad importance of GAG-mediated inflammation on cartilage pathology in the MPS diseases. Moreover, the positive effects of PPS observed in the tracheas were similar to those previously obtained using anti-TNF-alpha antibody therapy [9].

MicroCT analysis revealed modest improvements in the trabecular bone density and architecture of the femurs and vertebrae following PPS treatment. In contrast, no improvements were observed in the cortical bone, and no increases in femur length were found. In addition, PPS modestly restored the disc height index (i.e., disc height divided by adjacent vertebral length), disc height, torsional NZ length and NZ apparent modulus in the spines of MPS VI rats. These changes are suggestive of improved spinal instability, which is a critical problem in MPS patients that leads to spinal stenosis and cord compression. Slight improvements in vertebral failure load and stiffness also were observed in PPS-treated rats. The torsional test of the spine is a direct measurement of collagen integrity, and the improvement in torsional biomechanics with PPS also is suggestive of improved collagen integrity. Reduced intervertebral disc height in PPS treated animals is further supportive of improved collagen integrity. The relatively small improvements in geometry and spinal biomechanics with PPS treatment was anticipated since in this study PPS was used to inhibit TNF-alpha without addressing the underlying GAG storage. The above microCT and biomechanical changes also were only found in a subset of the treated rat, in contrast to the other studies performed in which all rats responded to varying degrees. The differential response in the spines and femurs could have been due to the limited bioavailability of the drug and/or differential intake through the drinking water by individual rats.

To investigate the possibility that PPS treatment might have altered the residual N4GNS activity in the tissues of the MPS VI rats, we prepared extracts and measured the N4GNS activity in vitro, and did not see any changes (Table S1). This is consistent with the fact that we did not see significant reductions in the GAG tissue levels (Fig. S1). Thus, it appears that PPS does not directly interact with N4GNS and is metabolized effectively in the MPS VI rats.

One of the most striking and significant clinical observations in all of the treated rats was the effect on motility. The mechanistic basis of this observation is not completely understood, and it did not appear to correlate with the modest improvements observed by microCT in the femurs and spines. We hypothesize that PPS treatment likely led to a significant reduction in pain of the MPS animals, contributing to the increased motility. A reduction in pain also is the most prominent change in IC patients treated with PPS [10], [21]. There also were clear changes in the articular chondrocytes that suggested improved articular joint function, which together with improvements in bone morphology and spine biomechanics might be partly responsible as well.

Another striking observation in the treated MPS VI rats was the effect of PPS on the skulls, which was observed in each of the treatment groups. The skulls and snouts were longer, which could be quantified in radiographs and by microCT analysis, and the snouts were thinner as well. Other notable skull improvements observed by microCT were evident in the dentition, where a greater degree of alignment was seen between the mandibular and maxillary molars, correcting the MPS-associated malocclusions. Improved tooth mineral densities also were observed. Dentition abnormalities are a common finding in MPS patients [29].

As noted above, the positive effect of PPS on cartilage metabolism and disease has been the subject of investigation for many years, and the clinical findings in OA patients have been recognized for over a decade [19]–[20]. The drug also has been used extensively in animals to treat OA [13], [16], [30], and is available commercially for OA veterinary use. The molecular mechanisms leading to the improvement in cartilage disease are not entirely clear, although PPS has been shown to repress MMP expression and inflammation, as well as NF-kB activation. It also enhances proteoglycan synthesis, including the production of aggrecan and hyaluronan [17].

It has been further suggested that because of the vascular effects of the drug it could decrease the rate of subchondral bone necrosis and sclerosis. Recently, PPS has been shown to promote the proliferation and chondrogenic differentiation of adult human bone marrow-derived mesenchymal stem cells as well [18]. Thus, the positive effects of PPS on the skull and other bones in the MPS VI rats could be due to any of these effects, and our data clearly show improvements in inflammation and factors that promote chondrogenesis.

It is important to note that our study evaluated oral PPS treatment for MPS, whereas in the OA literature the drug is generally administered subcutaneously. It is reasonable to speculate that the effects of subcutaneous PPS administration on the skeletal disease in MPS may be better than oral administration due to greater drug bioavailability, although to our knowledge there have been no side-by-side comparisons published in the literature to investigate this hypothesis.

Also, although the current study focused on the non-neural effects of PPS in MPS VI rats, the drug has been shown to have neural effects as well. In experimental animals PPS has been administered intraventricular and shown to inhibit the formation of protease resistant prion proteins in the brain [31]. It also has reduced neural inflammation in patients with Creutzfeld-Jakob disease. Neural inflammation is an important component of many disorders, including MPS, and reduction in inflammation has positive effects in several neurological lysosomal storage disease animal models [32]. PPS also has been shown to protect the blood brain barrier against amyloid-beta induced toxicity, and as well as to protect against ischemia-related neuronal death [33].

We previously evaluated the effects of anti-TNF-alpha antibody therapy in the MPS VI rats [9]. We found positive benefits from this therapy, although the effects on the skull, dentition and motility were less than those observed here with PPS. In the future it will be important to evaluate the combination of ERT and PPS, and to determine the additive benefit of this drug. While in our previous studies ERT was effective at reducing systemic inflammation, it had little or no effect on the cartilage and bone disease in MPS VI rats, and limited effects on motility. This was improved by anti-TNF-alpha antibody therapy, and we hypothesize will be even more significantly enhanced by combined PPS treatment.

In conclusion, PPS improved mobility and organ system pathologies known to occur in MPS VI rats, and these improvements were suggestive of reduced inflammation and pai. Thus, we propose that PPS could be beneficial in many MPS and other lysosomal storage disease patients, alone and in combination with existing therapies. However, more studies are needed to validate the use of PPS in these disorders, and more research is necessary to investigate its mechanism(s). In addition, while the safety of PPS has been extensively demonstrated in patients with IC, the long-term safety in MPS patients, particularly children, remains to be documented.

## Materials and Methods

### Animals

The MPS VI rats have been previously described and used extensively [5]–[8]. They are due to a point mutation in the gene encoding NG4S, and represent a natural animal model for this disorder. A breeding colony was established from heterozygous mating pairs, and genotyping was performed on tail clip DNA using established method [34]. Euthanasia of rats was performed using carbon dioxide inhalation. All animal protocols were approved by the Mount Sinai Institutional Animal Care and Use Committee (protocol # 08–0108), and were performed in accordance with NIH guidelines.

### Treatment of MPS VI Rats with PPS

Three groups of female MPS VI rats were treated by adding sodium PPS (Swati Spentose PVT LTD, India) to the drinking water at different ages: 6 months (group 1), 1 month (group 2), and prenatally by providing it to pregnant mothers (group 3), n = 10/group. Powdered PPS was dissolved in the drinking water to attempt to achieve a dose of 25 mg/kg/day (based on the weight of the rats and the amount of water intake per day). This would correspond to a human dose of 4 mg/kg/day based on standard drug dose conversion data for rats [35]. For patients with IC, PPS is generally used at a dose of 6 mg/kg/day.

For the prenatal group 3, after birth the lactating mothers were continued on PPS, and then the weaned rats were maintained on the drug throughout the study. All rats were treated until they reached 9 months of age. Only female rats were used to avoid gender-specific differences, and all control rats (normal and untreated MPS VI littermates) were gender and age-matched.

At the end of the study the animals were euthanized and tissues were collected from the control and treated MPS VI rats and placed in either phosphate buffered saline for the isolation of articular chondrocytes, or fixed in neutral buffered 10% formalin (Sigma Chemical) for histology, microCT analysis, and/or immunohistochemistry (see below).

### Serum Immunoassays

Serum was collected monthly from the control and treated rats, and inflammatory cytokines were assessed by enzyme-linked immunosorbent assays (ELISAs) using rat ELISA kits according to the manufacturers’ protocols. For rat TNF-alpha we used catalog #RTA00 from R & D Systems (Minneapolis, MN), for RANTES catalog #KRC1031 from Life Technologies (Grand Island, NY), for TIMP-1 catalog #RTM100 from R & D Systems, and for MIP-1alpha catalog #EA-3008 from Signosis (Sunnyvale, CA). Derivatives of AGEs, *N* ^e^-carboxymethyl-lysine (CML) and methyglyoxal (MG), also were quantified in serum by ELISA assays using monoclonal antibodies validated against synthetic standards, CML-BSA and MG-BSA, based on high performance liquid chromatography and gas chromatography-mass spectroscopy [24]. All serum assays were performed in triplicate.

### Immunoblot Analysis

After euthanasia articular chondrocytes were obtained by sequential enzyme digestion of cartilage from the femurs of treated and control rats, and grown for 3 weeks in monolayer cultures in DMEM containing 10% fetal bovine serum. They were then collected by trypsinization, pelleted by centrifugation, and lysed for immunoblot analysis as previously described [8]. The following antibodies from Santa Cruz Biotechnology (Santa Cruz, CA) were used for immunoblotting: anti-ADAMTS-5 (sc-28887), anti-p38 (A-12) (sc-7972), anti-Cox-2 (C-20) (sc-175450), anti-TNF-alpha (sc-1348), and anti-beta-actin (sc-1615). In some cases anti-GAPDH (sc-25778) also was used as a loading control.

### Immunohistochemical Analysis

For immunohistochemistry, paraffin-sections were fixed with 4% paraformaldehyde/PBS, permeabilized with 0.5% Triton-X-100, blocked, and incubated overnight at 4°C with anti-TNF-alpha antibody (sc-1348, Santa Cruz Biotechnology). After several rinses with PBS, visualization was accomplished in the lung and spleen sections using a fluorescent secondary antibody, donkey anti-goat IgG-Cy-3 (711-165-152, Jackson Laboratory). Nuclei were stained with 1 mg/ml bis-benzimide Hoechst dye (Sigma-Aldrich) for 10 min, rinsed, and sections were mounted with an anti-bleaching mounting media. Slides were visualized and photographed with a confocal laser-scanning microscope (Carl Zeiss 510 Meta). Heart sections were incubated with anti-TNF-alpha antibody (see above) and stained using the Ultravision Detection System according to the manufacturer’s protocol (Lab Vision Corporation).

### Radiographic and microCT Image Acquisition

Full body radiographs were taken on all of the age- and sex matched control and treated rats at the end of the study using a 43805 X-ray system (Faxitron series, Hewlett-Packard). Morphological traits and tissue mineral density (TMD) also were measured from the skulls, L_2_ vertebra of the spines, and femurs on a subset of rats using an eXplore Locus SP Preclinical Specimen MicroComputed Tomography (microCT) system (GE Healthcare, London, Ontario, Canada). Spine and femur microCT was carried out on 4–6 animals each of normal, untreated MPS VI, and PPS-treated group 2. For the skull, microCT was performed on 4–6 animals each from the control and treatment groups 2 and 3.

#### Vertebra

For radiographical analysis, lateral radiographs were taken for all L5–6 motion segments on all of the control and treated rats at the end of the study using a 43805 X-ray system (Faxitron series, Hewlett-Packard). MicroCT analysis was carried out on a subset of animals (see above). Intervertebral distances and vertebral lengths were measured, and the disc height index (DHI) also was determined by normalizing the intervertebral distance to adjacent vertebral lengths. L2 vertebra microCT images were reconstructed with a 12 µm isotropic voxel size. All scans were carried out at the end of treatment (9 months of age). Control rats were female and aged-matched. Regions of cortical and trabecular bone were manually separated and thresholded as previously described [36]. Cortical bone was analyzed for cortical thickness (CtTh), cortical area (CtAr), and tissue mineral density (Ct. TMD). Trabecular traits included bone volume density (BV/TV), trabecular thickness (TbTh), trabecular spacing (TbSp), trabecular number (TbN), and trabecular tissue mineral density (Tb TMD). All bone traits were assessed using Microview Advanced Bone Analysis software (v2.1.2; GE Healthcare). Total cross-sectional area (TtAr) was measured in 2D and averaged for all transverse sections along the height of the vertebral body. Vertebra height was calculated as the average vertebral body height at the center of the bone.

#### Femurs

Whole femurs from age-matched normal, untreated MPS VI and treatment group 2 rats were reconstructed with a 48 µm isotropic voxel size in order to measure femoral length. A region of the mid-diaphysis, beginning proximally just below the third trochanter, and ending distally at the appearance of the metaphysis was created. The analysis region was not restricted by measured size to accommodate variances in bone length. The mid-diaphysis was reconstructed with a 12 µm isotropic voxel size to determine cortical bone traits. Cortical thickness (CtTh), cortical area (CtAr), and cortical tissue mineral density (Ct TMD) were assessed using Microview Advanced Bone Analysis software (v2.1.2; GE Healthcare). Total area (TtAr) was calculated by averaging the cross-sectional area from all 2D images in mid-diaphysial section of bone. Additionally, a metaphysis region extending 6 mm proximally from the physis was selected for both cortical and trabecular analyses, including trabecular bone volume fraction (BV/TV), trabecular thickness (TbTh), trabecular spacing (TbSp), trabecular number (TbN), trabecular tissue mineral density (Tb TMD), as well as cortical thickness (CtTh), cortical area (CtAr), and cortical tissue mineral density (Ct TMD).

#### Skulls

Whole skulls from age-matched normal, untreated MPS VI and treatment groups 2 and 3 were reconstructed with an 88 µm isotropic voxel size. Regions of interest (ROI) were created for the whole skull and the lower incisors. For each ROI tissue mineral density (TMD) was calculated. Further linear measurements were taken to quantify differences in skull size and to characterize changes. Left and right eye orbit width, nose bridge width, snout length, and skull width. Additionally, the masseteric component was characterized by measuring the masseteric length (from anterior end of the masseteric crest to the end of the angular process), the mandibular height (from the posterior edge of the coronoid process to the end of the angular process) the sagittal molar misalignment (i.e. the distance between the molars from the anterior and posterior sides), and the inner-molar width (the smallest distance between each pair of molars). The respiratory component was characterized by measuring the nasal opening height (mid-sagittal view) and width (transverse view), the zygomatic width (the distance between the anterior roots of the zygomatic arch), and the nasal length (from the nasion to the rhinion). The neurocranial component was characterized by measuring the neurocranial length (Inferior edge of occipital bone to frontoparietal suture), the occipitosphenoidal length (parietal-occipital suture to inner sphenoidal suture), the interparietal width (greatest distance between parietal-interperial sutures), and the neurocranial width (distance between posterior zygomatic roots).

### Biomechanical Assessments of the Spine

Motion segment biomechanical properties were assessed via torsional experiments using an AR 2000ex rheometer (TA Instruments, New Castle, DE). The specimens were tested using a rotation-controlled approach with 2 loading stages: 1) equilibration (−1.875 N for 30 minutes) as a baseline for the torsional test [37], and 2) cyclic rotation test (±10° in both directions at 1 Hz). Ten degrees of rotation was chosen to insure both neutral zone (NZ) and linear region characteristics of the disc were included [38].

The data from the last cycle was used for analysis. Both neutral zone (NZ) and linear region characteristics were determined. The NZ length and stiffness were identified using the mathematical approach proposed by Smit et al. [39]. The linear stiffness was defined as the average of the slopes of the lines fitting between 50% and 100% of the minimum and maximum torque. The torque range also was determined and defined as the total range of torque applied over the span of 10° angular displacement in both directions. Geometric differences were taken into account in calculating the IVD biomechanical behaviors. The NZ and linear torsional stiffness (K, Nmm/°) were normalized to disc height (h, mm) and polar moment of inertia (J, mm4) using GAPP = Kh/J, where GAPP is the apparent shear modulus for NZ and linear regions. All analyses procedures were performed using Matlab software (R2011a, MathWorks Inc., Natick, MA).

### Rotarod Analysis

All rats were assessed and compared at the end of the study; i.e., 9 months of age. Rats were primed on the rod for 2 consecutive days prior to the actual recording. The rotarod was set at increasing speeds from 10 to 40 rpm over 3 minutes, and an average of the latency to fall off from the rod was recorded. Results were analyzed by one-way analysis of variance (ANOVA) with the variable group. In addition, videos were taken of the rats to document appearance and mobility in open space.

### Data Presentation and Statistical Analyses

Ten rats were enrolled in each of five groups (3 PPS treatment groups, untreated MPS VI and normal). MicroCT analysis was performed on a subset of these animals (as indicated above). In vitro assays (e.g., ELISAs) and rotarod analyses were performed on all of the rats and replicated at least 3 times. For western blots and microscopy, at least 5 rats from each group were studied; representative images are shown from each group. For statistical analyses the data between 2 groups were subjected to student’s t-test analysis, one-way analysis of variance (ANOVA) with the variable group, multivariate analyses of variance (MANOVAs) followed by post hoc Bonferroni adjustments. The results were considered significant at P<0.05. Statistics were performed using Sigma Stat 3.1 (Systat Software).

## Supporting Information

Figure S1 **Tissue and urine GAGs in PPS-treated MPS VI rats.** Tissue and urine GAGs were compared in the three groups of PPS-treated MPS VI rats (N = 10/group). Spleen, heart, kidney, liver and urine were collected when the animals were all 9 months of age. Hatched column, normal rat; white column, untreated MPS VI rat; light grey, MPS VI rat treated with PPS at 3 months of age for 6 months (group 1); dark grey, MPS VI rat treated with PPS at 1 month of age for 8 months (group 2) and black column, MPS VI rat treated with PPS prenatally and continued for 9 months (group 3). *P<0.05 comparing treated to untreated MPS VI rats.
(TIFF)

Table S1 **NG4S activities in organs from PPS-treated MPS VI rats.** NG4S activities where measured in the homogenates from the liver, kidney, heart, and spleen of PPS-treated MPS VI rats (all 3 groups, N = 30 total). Numbers shown represent total activities from all 3 groups. The activities did not vary between the 3 groups irrespective of when the PPS was initiated.
(DOC)

Movie S1 **Movies S1–S3 are movies of untreated and PPS-treated 9-month old MPS VI rats.** Movie S1 shows the typical appearance of an untreated adult MPS VI rat with an ungroomed coat and slow abnormal gait.
(MOV)

Movie S2 **Movies S1–S3 are movies of untreated and PPS-treated 9-month old MPS VI rats.** Movies S2–S3 show two MPS VI rats that were treated at one-month of age for 8 months (group 2). The treated MPS VI rats could stand on their hind limbs and were much more alert and mobile than their untreated littermates, and also had much smoother coats indicative of improved grooming.
(MOV)

Movie S3 **Movies S1–S3 are movies of untreated and PPS-treated 9-month old MPS VI rats.** Movies S2–S3 show two MPS VI rats that were treated at one-month of age for 8 months (group 2). The treated MPS VI rats could stand on their hind limbs and were much more alert and mobile than their untreated littermates, and also had much smoother coats indicative of improved grooming.
(MOV)
