# Supplementary figures and images for: Pentosan Polysulfate: A Novel Therapy for the Mucopolysaccharidoses
Source: PLoS One. 2013 Jan 24;8(1):e54459. doi: 10.1371/journal.pone.0054459 (PMC3554761; doi:10.1371/journal.pone.0054459)

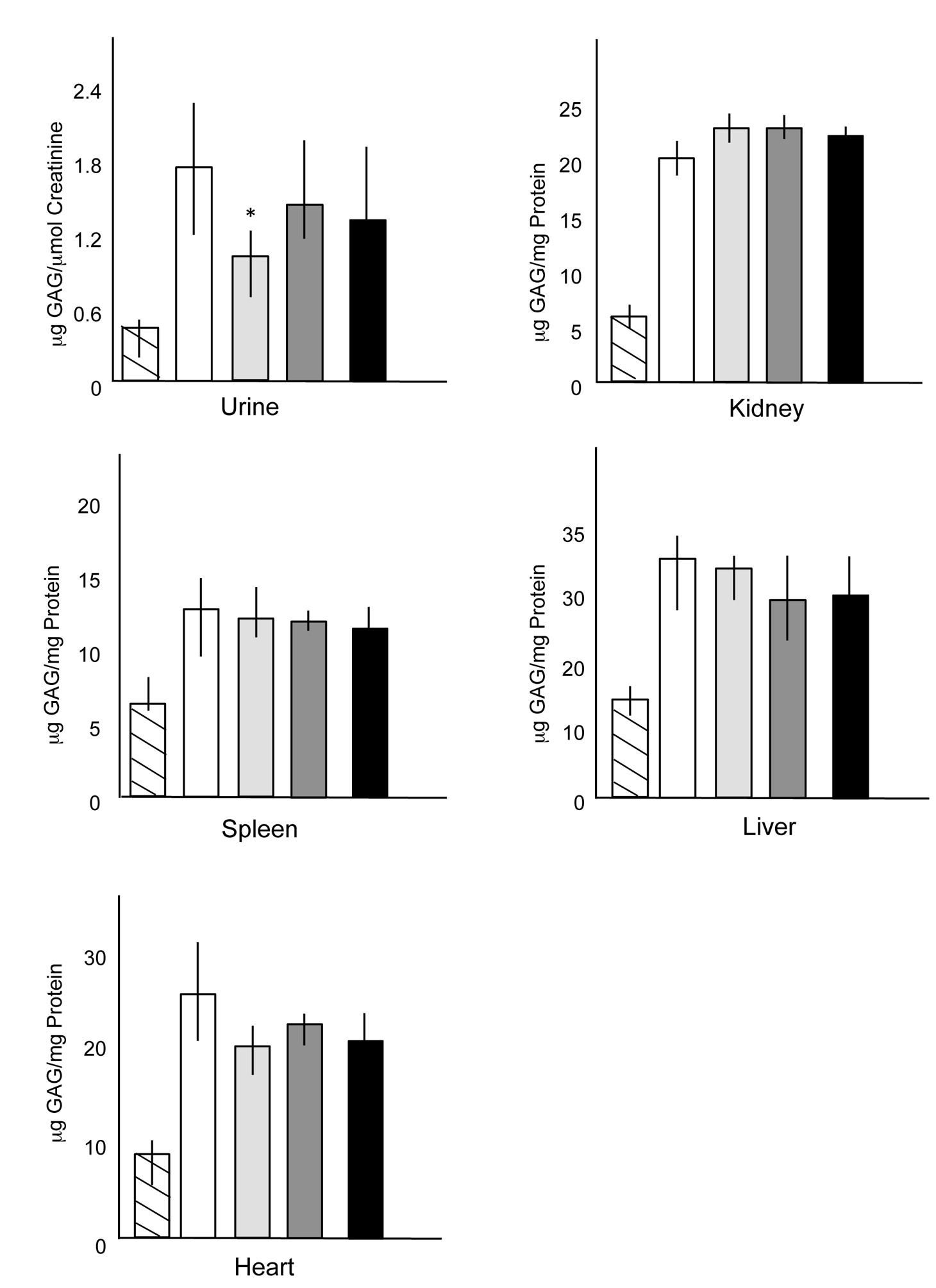

Supplement: Figure S1 — Tissue and urine GAGs in PPS-treated MPS VI rats. Tissue and urine GAGs were compared in the three groups of PPS-treated MPS VI rats (N = 10/group). Spleen, heart, kidney, liver and urine were collected when the animals were all 9 months of age. Hatched column, normal rat; white column, untreated MPS VI rat; light grey, MPS VI rat treated with PPS at 3 months of age for 6 months (group 1); dark grey, MPS VI rat treated with PPS at 1 month of age for 8 months (group 2) and black column, MPS VI rat treated with PPS prenatally and continued for 9 months (group 3). *P<0.05 comparing treated to untreated MPS VI rats. (TIFF) [file pone.0054459.s001.tiff]
