# Supplementary material for: Pentosan Polysulfate: A Novel Therapy for the Mucopolysaccharidoses
Source: PLoS One. 2013 Jan 24;8(1):e54459. doi: 10.1371/journal.pone.0054459 (PMC3554761; doi:10.1371/journal.pone.0054459)
Supplement: Table S1 — NG4S activities in organs from PPS-treated MPS VI rats. NG4S activities where measured in the homogenates from the liver, kidney, heart, and spleen of PPS-treated MPS VI rats (all 3 groups, N = 30 total). Numbers shown represent total activities from all 3 groups. The activities did not vary between the 3 groups irrespective of when the PPS was initiated. (DOC) [file pone.0054459.s002.doc]

**Table S1.** NG4S activities in organs from PPS-treated MPS VI rats

|  | Liver | Kidney | Heart | Spleen |
| --- | --- | --- | --- | --- |
| Normal | 58.0 +/- 14.0 | 475.0 +/- 20.0 | 74.3 +/- 6.0 | 576.1 +/- 90.0 |
| MPS | 4.0 +/- 0.3 | 2.8 +/- 0.4 | 2.3 +/- 0.9 | 5.1 +/- 0.3 |
| MPS + PPS | 5.8 +/- 0.5 | 2.8 +/- 0.2 | 2.4 +/- 0.1 | 5.2 +/- 0.2 |

N4GS activity expressed as nmol/hr/mg of protein
